# Œdème papillaire isolé révélant une sarcoïdose oculaire: Isolated papillary edema revealing ocular sarcoidosis

**DOI:** 10.11604/pamj.2019.32.132.18253

**Published:** 2019-03-20

**Authors:** Yasmine Chaoui Roqai, Yousra Ajhoun, Nisrine Laaribi, Soukaina Belfaiza, Yassine Mouzari, Fouad El Asri, Karim Reda, Abdelbarre Oubaaz

**Affiliations:** 1 Service d'Ophtalmologie, Hôpital Militaire d'Instruction Mohammed V, Faculté de Médecine et de Pharmacie Université Mohammed V, Rabat, Maroc

**Keywords:** Œdème papillaire, sarcoïdose oculaire, corticoïdes, Papillary edema, ocular sarcoidosis, corticosteroids

## Abstract

La sarcoïdose est une granulomatose multisystémique d'étiologie inconnue, défini par la présence de granulomes épithélioïdes et gigantocellulaires, sans nécrose caséeuse. La sarcoïdose oculaire se manifeste essentiellement par une uvéite antérieure granulomateuse et bilatérale. L'atteinte postérieure au cours de la sarcoïdose oculaire est rare et se manifeste par des périphlébites ou une atteinte choroïdienne. L'œdème papillaire isolé au cours est un tableau atypique d'où la particularité de notre observation. L'atteinte postérieure est un élément de mauvais pronostic menaçant le pronostic visuel, une prise en charge rapide en collaboration avec les pneumologues par corticothérapie systémique permet d'améliorer le pronostic visuel et limiter les complications.

## Abstract (en)

Sarcoidosis is a multisystem granulomatous disorders of unknown cause, characterised by the presence of epithelioid granulomas and giant-cell granulomas without caseous necrosis. Ocular sarcoidosis mainly manifests as bilateral anterior granulomatous uveitis. The involvement of the posterior segment in patients with ocular sarcoidosis is rare and manifests as periphlebitis or choroidal invasion. Isolated papillary edema is uncommon; hence the peculiarity of our case study. Posterior involvement is a prognostic indicator of poor outcome, threatening visual acuity. Early management, in close cooperation with pneumologists and based on systemic corticosteroid therapy, improves visual outcome and reduces complications.

## Introduction

La sarcoïdose est une granulomatose multisystémique d'étiologie inconnue, défini au sein de l'organe atteint, par la présence de granulomes épithélioïdes et gigantocellulaires, sans nécrose caséeuse. La localisation oculaire est révélatrice de la maladie dans 10 à 20% des cas [1]. Elle se manifeste essentiellement par une uvéite antérieure granulomateuse et bilatérale. Nous rapportons le cas d'une patiente présentant un œdème papillaire isolé unilatéral révélant une sarcoïdose.

## Patient et observation

**Figure 1:**
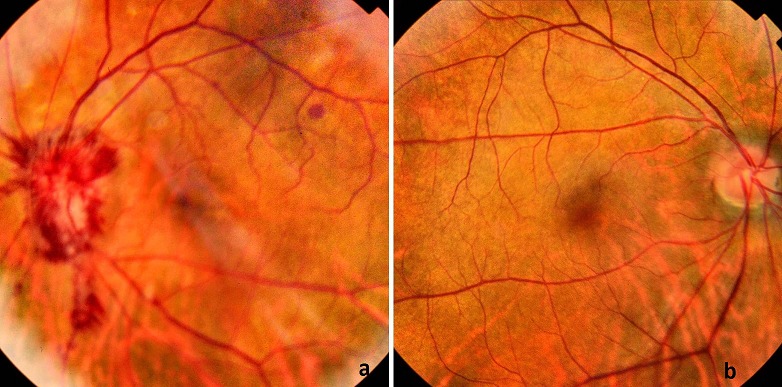
a) fond de l'œil gauche montrant un œdème papillaire avec hémorragies en flammèche; b) fond de l'œil droit normal

**Figure 2:**
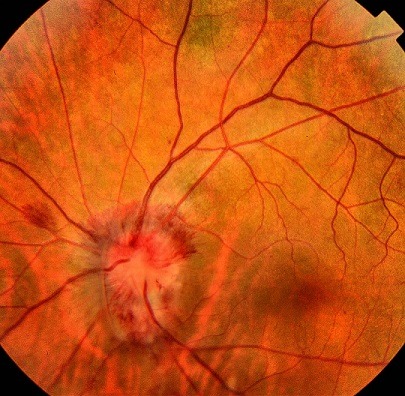
Fond d'œil à J6 du traitement régression de l'œdème papillaire et des hémorragies

Nous rapportons le cas d'une patiente âgée de 47 ans, forte myope, suivie en rhumatologie pour ostéoporose ayant présenté une baisse d'acuité visuelle brutale de l'œil gauche, sans autres signes associés. L'examen ophtalmologique de l'œil gauche retrouve une acuité visuelle corrigée à 1/20^ème^, l'examen des annexes et du segment antérieur est normal, tonus oculaire normal. Le fond d'œil retrouve un œdème papillaire stade III (aigu constitué), avec hémorragies en flammèche. L'examen ophtalmologique de l'œil droit était sans particularités. L'angiographie de l'œil gauche montrait un œdème papillaire isolé sans périphlébites associées (Figure 1), l'*optical coherence tomography* (OCT) papillaire altéré sur les quatre quadrants et un champ visuel altéré. Un bilan en urgence a été demandé révélant une enzyme de conversion à l'angiotensine élevée 116U/L, l'intradermo-réaction (IDR) à la tuberculine négative, une présence d'adénopathies hilaires à la radiographie thoracique, un aspect évocateur de sarcoïdose au scanner thoracique, la biopsie bronchique a objectivé la présence de granulomes épithélioïdes et gigantocellulaires, sans nécrose caséeuse. Une neurosarcoïdose a été éliminé devant la normalité de l'examen neurologique, de la ponction lombaire et de l'IRM orbito-cérébrale. Le diagnostic de sarcoïdose oculaire a été posé, en concertation avec le service de pneumologie, devant la preuve histologique et l'appui des examens complémentaires réalisés, même si le tableau clinique est atypique. Face au pronostic visuel menacé, la patiente a été mise sous bolus de solumédrol 1g/j pendant 3 jours, puis relais par voie orale à la dose de 1mg/kg/j. L'évolution a été marqué par une bonne amélioration clinique à J6 du traitement: augmentation de l'acuité visuelle à 6/10^ème^, régression de l'œdème papillaire et diminution des hémorragies (Figure 2).

## Discussion

L'atteinte ophtalmologique est fréquente au cours de la sarcoïdose et souvent révélatrice. L'âge de survenue est entre 20 et 40 ans, un deuxième pic est possible chez la femme en périménopausique. La manifestation la plus fréquente est une uvéite antérieure bilatérale granulomateuse. L'uvéite intermédiaire est retrouvée dans 10 à 20% des cas. L'atteinte postérieure est beaucoup plus rare, se manifeste essentiellement par des périphlébites segmentaires ou focales localisées au pôle postérieur ou en périphérie rétinienne [2], associé ou non à une atteinte choroïdienne à type de granulomes choroïdiens évolutifs jaunâtres, sous-rétiniens, souvent multiples, bilatéraux [3,4] localisés dans la région maculaire; l'œdème maculaire cystoïde, les granulomes papillaires ou juxta-papillaires. L'œdème papillaire est toujours associé à une hyalite et/ou à des périphlébites, le cas de notre observation est particulier, l'œdème papillaire était isolé sans autres signes oculaires. Les critères cliniques pour poser le diagnostic de sarcoïdose oculaire sont la présence de: précipités rétrodescemétiques granulomateux et/ou nodules iriens, trabéculite et/ou synéchies antérieures périphériques, opacités vitréennes en œufs de fourmi ou collier de perles, lésions choroïdiennes périphériques multiples, périphlébites segmentaires et/ou nodulaires et/ou macro-anévrysme, nodules ou granulome de la papille, bilatéralité de l'atteinte. Les critères paracliniques sont: la négativité de l'IDR à la tuberculine, élévation de l'enzyme de conversion de l'angiotensine, adénopathies hilaires bilatérales sur la radiographie du thorax, anomalies des ASAT, ALAT, γGT, aspect évocateur de sarcoïdose au scanner thoracique [1]. Les critères cliniques de diagnostic de sarcoïdose oculaire n'étaient pas présents chez notre patiente, le diagnostic a été posé devant les critères paracliniques ce qui fait la particularité de notre observation. Des complications sévères peuvent survenir: cataracte, glaucome secondaire, œdème maculaire, hémorragie intravitréenne sur une néovascularisation rétinienne. La complication oculaire la plus redoutable chez notre patiente est l'atrophie papillaire à long terme. Les facteurs de mauvais pronostic visuel sont [5,6]: âge avancé, patient mélanoderme, sexe féminin; maladie systémique chronique, atteinte du segment postérieur, retard de prise en charge supérieur à un an, acuité visuelle initiale basse, lésions rétiniennes périphériques, œdème maculaire ou une neuropathie glaucomateuse. Notre patiente a des éléments de mauvais pronostic: le sexe féminin, l'acuité visuelle initiale basse, l'atteinte papillaire ce qui rend le pronostic visuel menacé; la rapidité de prise en charge a permis la récupération visuelle, mais un suivi rapproché devant toute rechute est nécessaire. Le traitement de référence de la sarcoïdose est la corticothérapie [7]. Les bolus de méthylprednisolone sont réservés aux formes sévères menaçant le pronostic visuel. Son utilisation pour notre patient est justifiée. Les doses utilisées vont de 500 mg à 1 g/j pendant 3 jours; relayé par une corticothérapie par voie orale à la dose de 1mg/kg/j pendant 4 à 6 semaines sous surveillance, puis baissé par paliers réguliers de 10 mg tous les 10 jours jusqu'à demi-dose, avec un palier de 2 à 3 semaines à ce stade, puis de 5 mg tous les 10 jours jusqu'à une dose seuil autour de 10 mg, à adapter toujours en fonction de l'aspect clinique des lésions [1]. Si cortico-résistance on peut traiter par immunosuppresseurs.

## Conclusion

L'atteinte ophtalmologique est fréquente au cours de la sarcoïdose et souvent révélatrice. Les uvéites antérieures sont les plus fréquentes, et de bon pronostic. La forme postérieure se manifeste essentiellement par des périphlébites, ce cas particulier est caractérisé par un œdème papillaire isolé unilatéral révélateur de la maladie et menaçant le pronostic visuel. La prise en charge se fait conjointement avec les pneumologues et repose sur une corticothérapie au long cours et une surveillance régulière.

## Conflits d’intérêts

Les auteurs ne déclarent aucun conflit d'intérêts.
